# On Sympathy

**Published:** 1825-03

**Authors:** John Jones


[To be continued.]

---

### Art. IX.—*On Sympathy.* By John Jones, Esq.

This term, when used in reference to disease, implies that power by which parts are affected at a distance from the original seat of complaint. Its laws are at present but obscurely understood, and our knowledge of diseases which are under its influence is consequently imperfect. Some of its phenomena, however, appear to admit of a plausible solution, by considering them, like fever, as depending equally on the nervous and vascular systems.

The following division is founded on the preceding doctrines, and accords with the phenomena which occur.

1. Sympathy of Congestion.
2. ——— of Excitement.
3. ——— of Exhaustion.

1. *Sympathy of congestion.*—When a considerable organ is affected with venous congestion, nervous energy is not duly developed in that organ, and an accumulation of *materia vitæ diffusa* in the *un*congested vessels is the consequence. This may excite re-action, producing a slight increased vascular action, or actual inflammation, in the vicinity of the part originally affected, or in more distant parts, according to the peculiar susceptibility for morbid action from such a cause. Thus, the extremities are affected in gout, when the cause of the disease consists in a congestive state of the abdominal viscera. The brain is often sympathetically affected with the primæ viæ; and this connexion, there is reason to believe, is sometimes the cause of hydrocephalus. A striking instance, illustrative of this fact, I witnessed some time since, in the dissection of a child which had been long affected with tabes, and a few days previous to its death was attacked with symptoms of hydrocephalus, and died comatose. The liver was of a livid colour, much diseased in its structure, and the tubuli biliferi were gorged with black bile. Part of the intestinum ilium was greatly thickened, and formed a narrow stricture, extending some inches. Sanguineous effusion, to a considerable extent, had taken place on the surface of the brain. The adhesions between the dura mater and cranium were unusually strong; the cerebral substance was remarkably vascular, and the ventricles were distended with water.

Many instances of insanity are doubtless the effect of a congestive state of the abdominal viscera; and thus it is that purgatives are so often beneficial in such cases, when timely and perseveringly administered. It is true that insanity frequently appears to originate in the brain, and to depend on disorganisation of some part of that important organ. But even such disorganisation might be the effect of increased circulation, produced sympathetically with a congestive state of the abdominal viscera. What was at first an effect, if allowed to continue, becomes a cause, and confirmed insanity is too frequently the consequence.

When the internal organs are in a state of congestion, their functions are performed imperfectly, and their secretions consequently depraved. This is particularly remarkable in children. Their secretions are much more abundant than in adults; and susceptibility for morbid action in them is also greater. Most of their diseases are attributable to a vitiated state of the intestinal secretions: this is particularly the case with respect to cutaneous affections, to which they are so peculiarly subject. Instead of being the cause of such diseases, it appears more correct to consider the morbid state of the secretions as the primary effect of venous congestion in the secreting organs, and this latter, therefore, as the real cause of the disease. It is principally by removing visceral congestions, and thus enabling the obstructed organs duly to perform their functions, that emetics and purgatives are so often beneficial in removing disease, and not merely by evacuating the vitiated contents of the stomach and intestines. It is absurd to suppose that they should require such a system of purgation, as recommended by Dr. J. Hamilton, merely for their full evacuation.

2. *Sympathy of excitement*—exists when parts at a distance from the one originally affected are simultaneously excited into painful action. Thus, pain in the shoulder accompanies hepatitis; pain in the glans penis during micturition occurs sympathetically with the irritation of a stone in the bladder; the testicles are painfully affected by the passing of a stone from the kidneys. If the primary irritation be considerable, inflammation occurs, and the vascular system is thrown into inordinate action, constituting sympathetic fever. That connexion which exists between the liver and skin, which Dr. Johnson has noticed, and calls cutaneo-hepatic sympathy, to which he attributes such extensive influence in the production of tropical diseases, is of this description. The stimulus of the solar heat, acting on the surface, excites the liver to proportional increased action, and thus lays the foundation of disease of that organ. The nausea and vomiting, which occur in most cases of acute inflammation of the abdominal viscera, is also attributable to this kind of sympathy.

Sympathy of excitement is invariably connected with increased arterial action, which is always accompanied with a proportional increased production of nervous energy, causing the part to be morbidly sensible. This appears not to be confined to the original seat of complaint, but extends to distant nerves, according to the particular connexion which exists between the nerves of the part affected, and those of more distant parts.

3. *Sympathy of exhaustion*—is characterised by diminished nervous energy, occurring as the consequence of unusual excitement. It is either general, as when vomiting and fainting are the effect of severe pain; or partial, as when an external irritation removes an internal one.

Nervous energy, like the blood from which it is derived, is distributed to every part of the body in certain proportions. Inflammation consists in arterial action, which, as before observed, is always accompanied with a proportional evolution of nervous energy; the equal distribution of which, inflammation has a direct tendency to destroy. In this manner we may account for those sympathetic actions depending on what Mr. Burns very properly calls sympathy of equilibrium; by which he explains the beneficial effects resulting from blisters, setons, &c. He conceives that in proportion to the excitement produced by them, is the inflammatory action weakened which they are intended to remove.

*Debility*—is the effect of diminished nervous energy; and, although generally a symptom of disease, might be considered as arising from two causes: 1, protracted inordinate arterial action; 2, venous congestion.

1. *Protracted inordinate arterial action.*—The various actions of life are necessarily accompanied with an expenditure of nervous energy, and nature has wisely guarded against the consequences of such expenditure, by ordaining sleep as the means of replenishment. Thus the vigour of the system, which is diminished by our daily avocations, is restored by the balmy influence of slumber. If, when in health, an individual has been exposed to great exhaustion from inordinate exertion, in proportion to the unusual expenditure of the animal powers, must the duration of his slumbers be protracted, in order to restore the system to its wonted vigour; although this is greatly under the influence of habit, and therefore some persons require less sleep than others to produce the same effect. If the increased action arises from the occurrence of fever, the debilitating cause operates during sleep, as well as when awake; and consequently there is no replenishment of vigour. The debility progressively increases, either till the fever subsides or there is a total expenditure of the powers of life.

Debility is often connected with an irritable state of the system, which has a considerable influence in modifying disease. Irritability, in reference to disease, might be defined—a peculiar susceptibility for impressions, by which pain is readily induced, or the system unusually agitated by the application of stimuli. Thus, some individuals are affected more easily than others by mental impressions. Local injuries, which in some persons will only be productive of the most trifling symptoms, will in others cause violent inflammation and its consequences, by which the system might become sympathetically affected with fever, convulsions, &c. This state may depend on constitutional predisposition, or it may be the effect of disease. It is invariably the consequence of habitual intemperance. About the time of puberty in females, it disposes to hysteria; it particularly characterises the period of infancy, and modifies most of the diseases to which children are subject. The irritation which accompanies local injuries is evidently produced by a determination of arterial blood to the part; and we find that, in proportion to the arterial action excited, is the accompanying susceptibility for pain. If, then, different degrees of morbid action, from slight local irritation to positive inflammation, are owing to corresponding degrees of impetus of arterial blood, it seems fair to infer that the morbid irritability of the system, which so often accompanies debility, depends also on arterial action. That this is the case in those diseases peculiar to infancy, is proved by that period being particularly marked by arterial plethora; and likewise, in adults, morbid irritability is invariably accompanied by an accelerated pulse. Females appear more subject to this state of the system than males. Their nervous system is doubtless more delicately constructed, which renders it naturally more susceptible of impressions, and more active in deriving its peculiar principle from the blood: their pulse is quicker than that of the males, and we find that most of their diseases are greatly modified by irritability.

Morbid irritability, therefore, may be considered as partly constitutional, connected with weak stamina; and partly the effect of increased expenditure of nervous energy, in connexion with arterial action, in whatever way produced, and which becomes evolved in greater proportions than is consistent with the healthy energies of the system.

In children, debility is not a consequence of arterial plethora, because the increased production of nervous energy attending such a state is instrumental in promoting the growth of the body, and consequently adds to its vigour.

2. *Debility from congestion.*—This is the most frequent cause of debility. It has already been explained in what manner it has a direct tendency to produce this effect,—viz. by impeding the due development of nervous energy. When such impediment is suddenly induced in the principal internal organs, idiopathic fever is the consequence, in which the system is attacked by two formidable enemies: the one depriving it of its energies, by increased arterial action; the other preventing their re-production, by congestion. If, then, venous congestion necessarily produces debility, and is the most frequent cause of such a state, this important fact becomes an admirable guide in the treatment of a very numerous class of diseases, and cannot fail being of great practical utility.

*Derby; February 8th, 1825.*

